# Circulating Inflammatory Biomarkers Level before Thrombolysis for Acute Ischemic Stroke Predicts Symptomatic Intracerebral Hemorrhage

**DOI:** 10.14336/AD.2022.0608

**Published:** 2023-02-01

**Authors:** Lingzhi Li, Ziping Han, Zhenhong Yang, Qingfeng Ma, Haiping Zhao, Rongliang Wang, Junfen Fan, Liyuan Zhong, Yue Hu, Ping Liu, Yangmin Zheng, Yumin Luo

**Affiliations:** ^1^Institute of Cerebrovascular Disease Research and Department of Neurology, Xuanwu Hospital of Capital Medical University, Beijing, China.; ^2^Beijing Geriatric Medical Research Center and Beijing Key Laboratory of Translational Medicine for Cerebrovascular Diseases, Beijing, China.; ^3^Beijing Institute for Brain Disorders, Capital Medical University, Beijing, China

Dear Editor

Recombinant tissue plasminogen activator (rtPA) remains the only therapeutic agent approved for salvaging the penumbra through reperfusion after acute ischemic stroke (AIS) [1]. It effectively dissolves thrombi within 4.5 h after stroke onset, but it increases the risk of cerebral hemorrhage and malignant cerebral edema in AIS patients [2]. Thrombosis and inflammation are highly intertwined contributors to the pathophysiologic processes of stroke. In the post-ischemic period, inflammatory signaling is shortly activated, and cytokines and chemokines participate in the inflammatory responses resulting in thrombus formation and resolution. Several clinical studies showed that alterations in the expression level of various inflammatory cytokines in blood and cerebrospinal fluid of stroke patients were closely associated with the severity and clinical worsening of AIS [3]. A multi-center clinical study found circulating amyloid protein, alpha-2 macroglobulin, and metalloproteinase 9 independently predicted the three-month poor outcomes in AIS patients treated with thrombolysis [4]. More evidence is needed to demonstrate the association between inflammatory biomarkers before rtPA administration and the outcomes of AIS. Ten differentially expressed circulating inflammatory biomarkers (CD40L, HGF, IL-1β, IL-10, IL-16, IL-2, IL-5, IL-2Rα, CCL20, and MMP1) between AIS patients and healthy controls were screened from a broad panel of 65 cytokines [5]. The aim of this study thus was to investigate the association between the ten circulating inflammatory biomarkers before rtPA treatment and stroke outcomes as assessed by symptomatic intracerebral hemorrhage (sICH) after stroke.

The study design was approved by the Ethics Committee of Xuanwu Hospital, Capital Medical University, and conducted complied with the principles of the Declaration of Helsinki. Written, informed consents were obtained from all patients or their legal representatives before enrollment. From November 2018 to May 2019, consecutive 496 patients were enrolled in the stroke center of Xuanwu Hospital of Capital Medical University. Finally, 242 AIS patients were enrolled in our analysis (Supplementay Table 1). sICH was defined according to the ECASS-III after stroke. All patients on admission underwent blood sampling as soon as possible. Plasma levels of CD40L, HGF, IL-1β, IL-10, IL-16, IL-2, IL-5, IL-2Rα, CCL20, and MMP1 were measured using a ProcartaPlex multiplex magnetic bead panel kit (Invitrogen, PPX-10). The data were then analyzed by the ProcartaPlex Analyst 1.0 software. All the data were analyzed with R studio 3.6.1 (Boston, MA), SPSS 26 (SPSS Inc, Chicago, IL), and GraphPad Prism 8.4.0 (GraphPad Software, La Jolla, CA). *p* < 0.05 was set as statistical significance. The least absolute shrinkage and selection operator (LASSO) regression was chosen to select a subset of useful predictors to construct the multi-biomarker scores from the ten candidate biomarkers, through five-fold cross-validation. The scores were defined as:

Score = *β*_0_ + *β*_A*_ biomarker A + *β*_B*_ biomarker B + *β*_c*_ biomarker C, etc., where β_A_, β_B_, β_c,_ etc represented regression β-coefficients for biomarkers A, B, and C, respectively, and β_0_ represented an intercept from the LASSO regression. The accuracy of the models with and without a multi-biomarker score was compared by calculating the areas under the curve (AUC) with the DeLong method. Improvement of discrimination and reclassification of the predictive models with the addition of multi-biomarker score was calculated by the integrated discrimination improvement (IDI) and categorical or continuous net reclassification improvement (NRI) indexes. We performed Gene Ontology (GO) and Kyoto Encyclopedia of Genes and Genome (KEGG) enrichment analysis of the biomarkers in the multi-biomarker score^*^ using the Database for Annotation, Visualization, and Integrated Discovery (DAVID).

**Figure 1.**
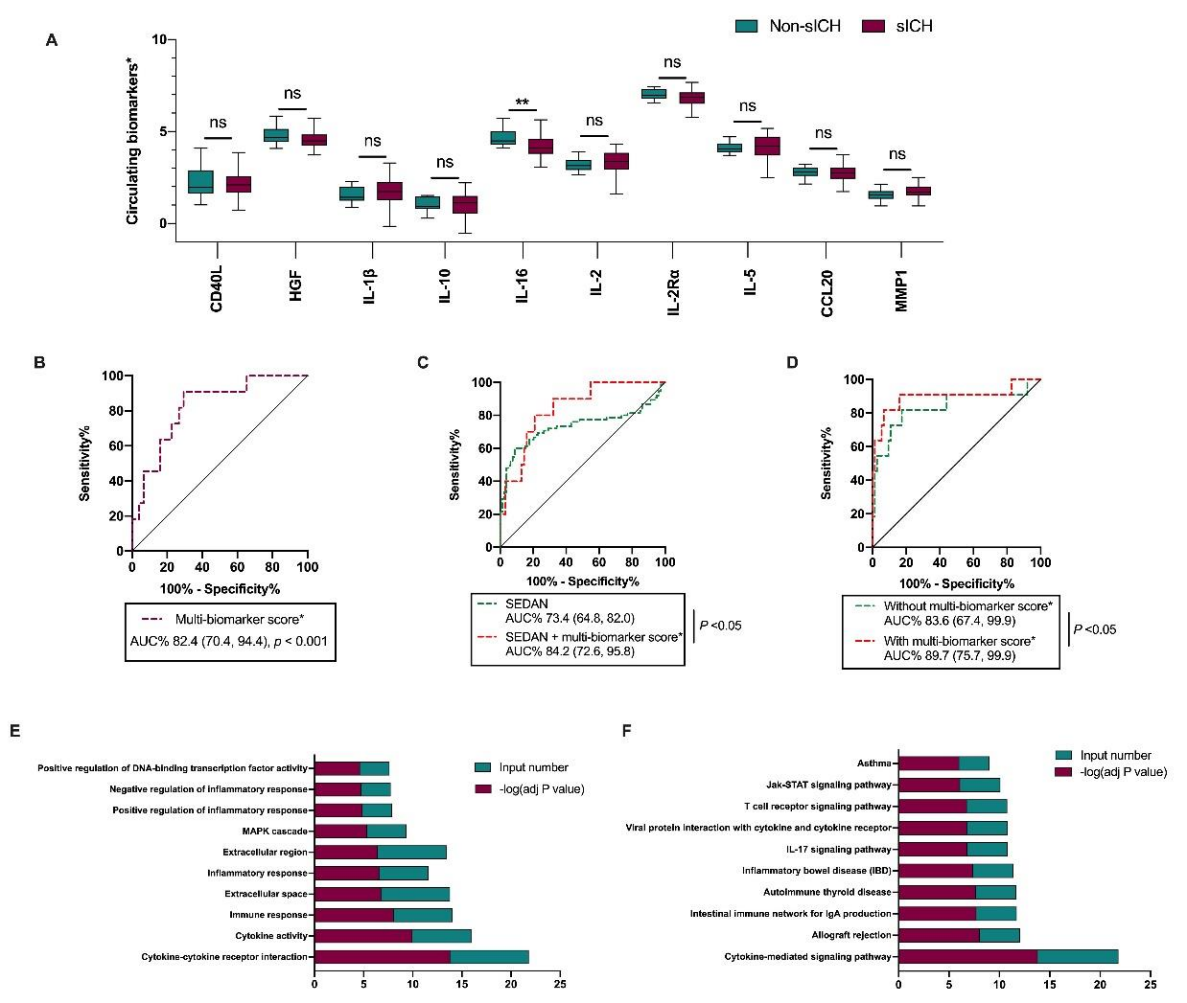
**Circulating inflammatory biomarkers before thrombolysis for predicting symptomatic intracerebral hemorrhage after acute ischemic stroke**. **(A)** Comparisons of circulating inflammatory biomarkers between patients with and without symptomatic intracerebral hemorrhage within 3 months after rtPA treatment. Y axis: Ln transformation of the ten circulating inflammatory biomarkers. Lines of the boxes represented medians, and error bars represented interquartiles. rtPA, recombinant tissue plasminogen activator. **(B)** ROC analysis of univariate regression models for symptomatic intracerebral hemorrhage within 3 months with the multi-biomarker score*. **(C)** ROC curves of multivariate models for symptomatic intracerebral hemorrhage within 3 months with (red) or without the multi-biomarker score* (green), adjusted by age, admission NIHSS score, atrial fibrillation, lymphocyte count, and total cholesterol. **D)** ROC curves of multivariate models involved SEDAN for symptomatic intracerebral hemorrhage within 3 months with (red) or without the multi-biomarker score* (green). ROC, receiver operating characteristic curve; SEDAN, a clinical prediction model to identify patients with sICH after rtPA treatment (composed of sugar, early infarct signs, hyperdense cerebral artery sign, age, and NIH Stroke Scale). **(E)** GO analysis of the variables in the multi-biomarker score*. **(F)** KEGG analysis of the variables in the multi-biomarker score*. Input number indicated the number of variables enriched; adj P value indicated the FDR-adjusted P value; GO, Gene Ontology; KEGG, Kyoto Encyclopedia of Genes and Genomes.

**Table 1:** Logistic regression analysis and additional predictive value of the model including the multi-biomarker score* for patients with sICH after receiving rtPA treatment (n = 91).

		sICH
	Clinical model	Clinical model + multi-biomarker score*
Logistic regression		
R^2^ (Cox & snell)	0.168	0.280
Age	OR = 1.018 (0.950-1.092), *p* = 0.607	OR = 1.031 (0.935-1.098), *p* = 0.752
Admission NIHSS score Atrial fibrillation Lymphocyte count Total cholesterol	OR = 1.185 (1.037-1.355), *p* = 0.013 † OR = 0.854 (0.102-7.115), *p* = 0.884 OR = 0.691 (0.314-1.516), *p* = 0.356 OR = 0.710 (0.362-1.395), *p* = 0.321	OR = 1.156 (0.990-1.349), *p* = 0.068 OR = 0.728 (0.076-7.015), *p* = 0.783 OR = 0.703 (0.288-1.718), *p* = 0.440 OR = 0.469 (0.193-1.138), *p* = 0.094
Multi-biomarker score* ≥ -1.129	-	OR = 3.075 (1.115-8.477), *p* = 0.030 †
ROC curve		
AUC, %	83.6	89.7
*p* Value	Ref.	0.037
IDI index, %		
Total IDI	-	17.0 (1.1, 32.9)
*p* Value	Ref.	0.036
NRI index, %		
Categorical NRI	-	35.0 (5.8, 64.3)
*p* Value	Ref.	0.019
Continuous NRI	-	129.5 (75.4, 183.5)
*p* Value	Ref.	< 0.001

† *p* < 0.05. Abbreviations: AUC = Area under the curve; IDI = Integrated discrimination improvement; NIHSS = NIH Stroke Scale; NRI = Net reclassification improvement; ROC = Receiver operating characteristic curve. multi-biomarker score* = -1.7398617061 - 0.0081435148*CD40L - 0.5497569604*IL-1β + 0.1563118707*IL-10 + 0.0009169552*IL-16 - 0.0039681473*IL-2 - 0.0002497288*IL-2Rα + 0.0305756400*IL-5 + 0.0726352949*CCL20 - 0.0040341082* MMP1

In the present study, we demonstrated an association between a panel of circulating inflammatory biomarkers and the occurrence of sICH of AIS patients received rtPA treatment. Among the 242 AIS patients, 102 patients received rtPA treatment. Between patients eligible for IV-rtPA or not, plasma concentration of the ten inflammatory biomarkers did not vary significantly (Supplementary Table 2). Among the 102 patients received rtPA treatment, 11 patients received bridging therapy were excluded. According to sICH after rtPA treatment, there’re 12 patients with sICH and 79 patients without (Supplementary Table 3), and a decreased IL-16 concentration was associated with sICH (Fig. 1A). We found that multi-biomarker score^*^ (including CD40L, IL-1β, IL-10, IL-16, IL-2, IL-5, IL-2Rα, CCL20, and MMP1) was an independent predictor for sICH after rtPA treatment (Table 1, Figure 1B, C). Particularly, multi-biomarker score^*^ added prognostic value to the SEDAN model for predicting sICH after rtPA administration (Table 2, Fig. 1D). Mounting preclinical studies showed that inflammatory cytokines were associated with blood-brain barrier disruption and hemorrhage transformation (HT) [6,7]. A genome-wide association meta-analysis of clinical trials involved reperfusion therapies implicated that inflammation and β amyloid aggregation-related pathways were related to HT [8]. Moreover, a cross-sectional study found that higher levels of circulating TNFR2 and myeloperoxidase out of a panel of 15 inflammation-associated biomarkers were associated with the presence of cerebral microbleeds [9]. What’s more, a prospective study involving 431 stroke patients without histories of cerebrovascular diseases also showed that higher circulating IL-6, high-sensitivity C-reactive protein (hsCRP), and IL-18 were associated with deep or lobar cerebral microbleeds [10]. Consistently, our study suggests that the multi-biomarker score^*^is predictive of the occurrence of sICH after rtPA treatment. Specifically, AIS patients with circulating multi-biomarker score^*^ ≥ -1.129 were predicted to suffer from an additional 3.075-fold risk (with confounders adjustment) to develop sICH after rtPA treatment. Additionally, entering the multi-biomarker score^*^ into the clinical variables significantly improved the AUC value, NRI, and IDI (Table 1). Several clinical prediction models to identify patients with sICH have been developed, including MSS, HAT, SEDAN, GRASPS, SITS, and SPAN-100 positive index. Among them, SEDAN (composed of blood sugar, early infarct signs, hyperdense cerebral artery sign, age, and NIH Stroke Scale) [11] showed the highest predictive power. Therefore, we calculated the SEDAN in our cohort of patients received rtPA treatments and generated the new model of SEDAN combined with the multi-biomarker score^*^. Compared with the SEDAN, the addition of the multi-biomarker score^*^ improved the AUC value, NRI, and IDI as well (Table 2). Bioinformatic analysis of these biomarkers in the multi-biomarker score^*^ suggests that they are significantly enriched in the biological processes including "Cytokine-cytokine receptor interaction", "Immune response", "Inflammatory response", "Positive regulation of inflammatory response", and "Negative regulation of inflammatory response"; and enriched in the KEGG pathways such as "Cytokine-mediated signaling pathway", "IL-17 signaling pathway", and "T cell receptor signaling pathway" (Figure 1E,F), which all supported the possible role of immune response and inflammation for sICH after rtPA treatment. The interaction of these inflammatory biomarkers and the action of inflammatory biomarkers on immune cells, shapes the progress of immune response in the ischemic brain, thereby determining the severity of neurological impairment and clinical prognosis of AIS patients [12]. Preclinical studies showed that IL-1β and IL-16 played pro-inflammatory roles after AIS, whereas IL-10, IL-2, and IL-2Rα played anti-inflammatory effects [13], and prognostic significance of CD40L, IL-1β, IL-10, IL-16, IL-2, IL-5, IL-2Rα, and MMP1had been implicated in clinical studies. Consistently, we here demonstrate that the multi-biomarker score^*^ composed of the nine biomarkers possess predictive value for the occurrence of sICH after AIS, and the possible mechanism of the interaction between these biomarkers warrants future investigation.

**Table 2:** Logistic regression analysis and additional predictive value of the model including the multi-biomarker score* for patients with sICH after receiving rtPA treatment (n = 91).

		sICH
	SEDAN score	SEDAN score + multi-biomarker score*
Logistic regression		
R^2^ (Cox & snell)	0.148	0.244
SEDAN	OR = 1.444 (0.676-3.082), *p* = 0.342	OR = 1.253 (0.517-3.036), *p* = 0.618
Multi-biomarker score* ≥ -1.129	-	OR = 3.496 (1.431-8.544), *p* = 0.006 †
ROC curve		
AUC, %	73.4	84.2
*p* Value	Ref.	0.021
IDI index, %		
Total IDI	-	19.2 (0.1, 38.3)
*p* Value	Ref.	<0.05
NRI index, %		
Categorical NRI	-	24.8 (-14.0, 63.7)
*p* Value	Ref.	0.210
Continuous NRI	-	84.5 (20.9, 148.2)
*p* Value	Ref.	0.009

† *p* < 0.05. Abbreviations: AUC = Area under the curve; IDI = Integrated discrimination improvement; NIHSS = NIH Stroke Scale; NRI = Net reclassification improvement; ROC = Receiver operating characteristic curve. multi-biomarker score* = -1.7398617061 - 0.0081435148*CD40L - 0.5497569604*IL-1β + 0.1563118707*IL-10 + 0.0009169552*IL-16 - 0.0039681473*IL-2 - 0.0002497288*IL-2Rα + 0.0305756400*IL-5 + 0.0726352949*CCL20 - 0.0040341082* MMP1

## Supplementary Materials

he Supplementary data can be found online at: www.aginganddisease.org/EN/10.14336/AD.2022.0608.
