# Supplementary material for: Circulating Inflammatory Biomarkers Level before Thrombolysis for Acute Ischemic Stroke Predicts Symptomatic Intracerebral Hemorrhage
Source: Aging Dis. 2023 Feb 1;14(1):9–13. doi: 10.14336/AD.2022.0608 (PMC9937699; doi:10.14336/AD.2022.0608)
Supplement: Supplementary file 1 — he Supplementary data can be found online at: www.aginganddisease.org/EN/10.14336/AD.2022.0608. [file AD-14-1-9-s.pdf]

SUPPLEMENTARY DATA

Circulating Inflammatory Biomarkers Level before Thrombolysis for Acute Ischemic Stroke Predicts Symptomatic Intracerebral Hemorrhage

Lingzhi Li[1], Ziping Han[1,2], Zhenhong Yang[1], Qingfeng Ma[1], Haiping Zhao[1,2], Rongliang Wang[1,2], Junfen Fan[1], Liyuan Zhong[1], Yue Hu[1], Ping Liu[1], Yangmin Zheng [1,2*], Yumin Luo[1,2,3*]

© 2022. Li L et al. Published online at http://www.aginganddisease.org/EN/10.14336/AD.2022.0608

Supplementary Table 1. Baseline characteristics of the study population according to the modified Rankin Scale [mRS] 0–1 at 3 months.

	Total (N=242)	Excellent outcome[a] (N=125)	Poor outcome (N=117)	*p* Value
**Demographic characteristics**				
**Age, y, mean (SD)**	65.0 (13.2)	63.0 (13.2)	67.1 (13.0)	0.014 †
**Female sex (%)**	65 (26.9)	32 (25.6)	33 (28.2)	0.755
**BMI, kg/m^2, median [IQR]**	25.4 [23.9, 27.4]	25.4 [23.8, 27.3]	25.7 [24.0, 27.3]	0.987
**Medical history**				
**Hypertension**	161 (66.5)	82 (65.6)	79 (67.5)	0.857
**Diabetes mellitus**	81 (33.5)	37 (29.6)	44 (37.6)	0.237
**Hyperlipemia**	67 (27.7)	43 (34.4)	24 (20.5)	0.023 †
**Coronary heart disease**	50 (20.7)	21 (16.8)	29 (24.8)	0.169
**Atrial Fibrillation**	44 (18.2)	14 (11.2)	30 (25.6)	0.006 †
**Recurrent stroke**	92 (38.0)	44 (35.2)	48 (41.0)	0.423
**Smoking habit**	61 (42.4)	33 (43.4)	28 (41.2)	0.918
**Stroke characteristics and treatment**				
**Admission NIHSS score**	5.0 [3.0, 11.0]	3.0 [1.0, 4.0]	11.0 [8.0, 16.0]	<0.001 †
**Onset-to-treatment time, h**	3.0 [1.5, 5.1]	2.5 [1.3, 3.9]	3.7 [1.9, 6.8]	<0.001 †
**rtPA treatment**	92 (38.0)	63 (50.4)	29 (24.8)	<0.001 †
**Clinical and laboratory findings**				
**Systolic blood pressure, mm Hg**	150.0 [138.5, 167.8]	151.0 [138.0, 170.0]	150.0 [140.0, 164.0]	0.305
**Diastolic blood pressure, mm Hg**	85.5 [78.0, 92.0]	85.0 [78.0, 93.0]	87.0 [78.0, 91.0]	0.769
**Serum glucose, mmol/L**	7.1 [5.9, 10.0]	6.8 [5.8, 8.9]	7.6 [6.1, 10.5]	0.050
**HbA1c, %**	6.0 [5.5, 7.3]	5.9 [5.4, 6.9]	6.1 [5.7, 7.4]	0.091
**Neutrophils, ×1,000/mm^3**	5.1 [3.9, 6.6]	4.6 [3.8, 5.9]	5.5 [4.0, 7.8]	0.002 †
**NLR**	2.9 [2.1, 5.6]	2.5 [1.9, 3.7]	4.2 [2.3, 7.6]	<0.001 †
**Platelet count, ×1,000/mm^3**	208.0 [171.0, 244.8]	215.0 [182.0, 257.0]	197.0 [160.0, 231.0]	0.010 †
**HYC, μmol/L**	15.5 [12.0, 19.2]	15.6 [12.0, 20.6]	15.2 [12.0, 18.2]	0.432
**TG, mmol/L**	1.4 [1.0, 2.4]	1.6 [1.1, 2.7]	1.3 [0.8, 2.0]	0.009 †
**TC, mmol/L**	4.5 [3.8, 5.4]	4.7 [3.9, 5.5]	4.4 [3.7, 5.2]	0.155
**HDL, mmol/L**	1.2 [1.0, 1.4]	1.2 [1.0, 1.4]	1.2 [1.0, 1.4]	0.613
**LDL, mmol/L**	2.7 [2.1, 3.4]	2.8 [2.1, 3.5]	2.7 [2.0, 3.3]	0.409
**Stroke etiology (TOAST), n (%)**				
**Large artery atherosclerosis**	137 (56.6)	68 (55.7)	69 (57.5)	0.883
**Small vessel occlusion**	68 (28.1)	42 (34.4)	26 (21.7)	0.059
**Cardioembolic**	12 (5.0)	3 (2.5)	9 (7.5)	0.131
**Other determined**	1 (0.4)	0 (0.0)	1 (0.8)	0.933
**Undetermined**	24 (9.9)	9 (7.4)	15 (12.5)	0.264
**Posterior circulation stroke**	37 (15.3)	17 (13.9)	20 (16.7)	0.680
**Biological measures, pg/ml**				
**CD40L**	8.4 [5.5, 13.7]	8.2 [5.3, 13.2]	8.6 [5.5, 13.9]	0.721
**HGF**	101.0 [77.0, 135.4]	94.7 [68.8, 117.7]	110.3 [84.5, 169.7]	0.001 †
**IL-1β**	5.2 [3.2, 8.8]	5.8 [3.4, 9.7]	4.8 [3.2, 8.2]	0.130
**IL-10**	2.9 [1.8, 4.6]	3.0 [1.7, 4.6]	2.8 [2.0, 4.6]	0.717
**IL-16**	68.8 [47.6, 108.7]	61.7 [47.1, 103.0]	71.8 [48.9, 118.2]	0.187
**IL-2**	26.9 [17.2, 41.2]	29.5 [19.4, 47.4]	24.3 [15.7, 36.9]	0.013 †
**IL-2Rα**	991.5 [719.8, 1407.7]	920.4 [682.5, 1257.1]	1070.5 [781.1, 1497.8]	0.032 †
**IL-5**	63.2 [35.1, 102.5]	66.5 [37.3, 112.3]	57.9 [34.9, 92.4]	0.194
**CCL20**	15.2 [11.2, 20.9]	15.2 [11.2, 20.9]	15.2 [11.3, 20.6]	0.979
**MMP1**	5.7 [4.5, 7.2]	5.9 [4.8, 7.3]	5.4 [4.3, 7.0]	0.113

[a] The excellent outcome was defined as a mRS score = 0-1 and the poor outcome was defined as a mRS score > 1.

Abbreviations: BMI = Body Mass Index; HbA1c = HemoglobinA1c; HDL = High density lipoprotein; HYC = Homocysteine; LDL = Low density lipoprotein; mRS = modified Rankin Scale; NIHSS = NIH Stroke Scale; NLR = Neutrophil-to-Lymphocyte Ratio; rtPA = recombinant tissue plasminogen activator; sICH = symptomatic intracerebral hemorrhage; TC = Total cholesterol; TG = Triglyceride; TOAST = Trial of ORG 10172 in Acute Stroke Treatment.

Data for continuous variables are described as mean (SD) (normally distributed variables) or as median [interquartile range] (nonnormally distributed variables), for categorical variables are described as n (%).

† $p < 0.05$.

© 2022. Li L et al. Published online at http://www.aginganddisease.org/EN/10.14336/AD.2022.0608

Supplementary Table 2. Comparisons of circulating biomarkers concentrations between patients eligible for IV recombinant tissue plasminogen activator (rtPA) or not.

	Total (N=242)	rtPA treatment (N=102)	Non-rtPA treatment (N=140)	*p* Value
CD40L, pg/ml	8.4 [5.5, 13.7]	8.0 [5.3, 12.1]	8.6 [5.7, 14.0]	0.430
HGF, pg/ml	101.0 [77.0, 135.4]	90.0 [71.2, 126.8]	103.7 [79.1, 138.6]	0.124
IL-1β, pg/ml	5.2 [3.2, 8.8]	5.4 [3.6, 8.3]	4.9 [3.1, 8.9]	0.447
IL-10, pg/ml	2.9 [1.8, 4.6]	3.1 [1.9, 4.1]	2.8 [1.7, 4.8]	0.832
IL-16, pg/ml	68.8 [47.6, 108.7]	69.5 [47.1, 103.6]	67.0 [48.3, 109.3]	0.742
IL-2, pg/ml	26.9 [17.2, 41.2]	27.6 [18.3, 45.0]	26.4 [16.1, 39.9]	0.312
IL-2Rα, pg/ml	991.5 [719.8, 1407.7]	962.6 [687.8, 1263.4]	1026.1 [762.8, 1520.0]	0.123
IL-5, pg/ml	63.2 [35.1, 102.5]	66.4 [45.0, 107.3]	60.8 [31.5, 98.3]	0.203
CCL20, pg/ml	15.2 [11.2, 20.9]	15.8 [11.5, 20.5]	15.2 [11.2, 20.9]	0.574
MMP1, pg/ml	5.7 [4.5, 7.2]	5.5 [4.5, 7.2]	5.9 [4.5, 7.3]	0.742

Note: Applicable data are numbers of arteries with percentages in parentheses.

Supplementary Table 3. Baseline characteristics of the patients received rtPA treatment according to the occurrence of symptomatic intracerebral hemorrhage.

	Total (N=91)	Non-sICH (N= 79)	sICH (N= 12)	*p* Value
**Demographic characteristics**				
Age, y, mean (SD)	62.9 (12.0)	61.8 (11.5)	69.9 (13.1)	0.029 †
Female sex (%)	23 (25.3)	18 (22.8)	5 (41.7)	0.296
BMI, kg/m^2, median [IQR]	25.4 [23.9, 27.4]	25.4 [23.9, 27.3]	26.2 [24.4, 29.6]	0.483
**Medical history**				
Hypertension	62 (68.1)	54 (68.4)	8 (66.7)	-
Diabetes mellitus	29 (31.9)	25 (31.6)	4 (33.3)	-
Hyperlipemia	45 (49.5)	40 (50.6)	5 (41.7)	0.788
Coronary heart disease	18 (19.8)	14 (17.7)	4 (33.3)	0.381
Atrial Fibrillation	11 (12.1)	7 (8.9)	4 (33.3)	0.049 †
Recurrent stroke	36 (39.6)	29 (36.7)	7 (58.3)	0.267
Smoking habit	36 (45.0)	32 (46.4)	4 (36.4)	0.769
**Stroke characteristics and treatment**				
Admission NIHSS score	5.0 [3.0, 6.5]	4.0 [3.0, 6.0]	10.0 [4.2, 16.5]	0.027 †
Onset-to-treatment time, h	2.4 [1.2, 3.5]	2.5 [1.2, 3.5]	2.1 [1.2, 3.3]	0.647
24h NIHSS score	3.0 [2.0, 5.0]	3.0 [2.0, 4.0]	8.0 [3.5, 13.2]	0.008 †
7day NIHSS score [b]	1.0 [1.0, 4.0]	1.0 [0.0, 3.0]	7.5 [2.5, 9.8]	0.004 †
**Clinical and laboratory findings**				
Systolic blood pressure, mm Hg	153.0 [140.0, 170.0]	152.0 [140.0, 170.0]	154.5 [142.8, 168.0]	0.930
Diastolic blood pressure, mm Hg	83.0 [77.0, 95.0]	83.0 [77.0, 96.0]	81.0 [75.5, 89.2]	0.259
Serum glucose, mmol/L	7.0 [6.0, 10.3]	7.0 [5.9, 9.9]	8.2 [6.5, 14.6]	0.281
HbA1c, %	6.1 [5.6, 7.9]	6.1 [5.5, 7.5]	6.2 [5.8, 9.1]	0.216
Neutrophils, ×1,000/mm^3	4.7 [3.7, 5.8]	4.6 [3.7, 5.7]	5.2 [4.1, 6.7]	0.418
Lymphocytes, ×1,000/mm^3	1.8 [1.2, 2.3]	1.8 [1.3, 2.3]	1.3 [0.8, 2.1]	0.044 †
NLR	2.5 [1.9, 4.2]	2.5 [1.7, 3.9]	3.2 [2.3, 6.6]	0.113
Platelet count, ×1,000/mm^3	217.0 [179.5, 260.0]	215.0 [182.5, 262.0]	228.5 [172.0, 242.0]	0.842
HYC, μmol/L	14.8 [11.5, 19.7]	15.6 [11.4, 20.6]	13.9 [12.9, 15.3]	0.278
TG, mmol/L	1.8 [1.2, 2.9]	1.8 [1.2, 2.8]	2.0 [1.0, 4.1]	0.977
TC, mmol/L	4.6 [3.9, 5.6]	4.7 [4.0, 5.6]	4.2 [3.5, 4.8]	0.049 †
HDL, mmol/L	1.2 [1.0, 1.3]	1.2 [1.0, 1.4]	1.1 [1.0, 1.2]	0.225
LDL, mmol/L	2.7 [2.2, 3.3]	2.7 [2.3, 3.6]	2.5 [2.1, 2.9]	0.195
**Stroke etiology (TOAST), n (%)**				
Large artery atherosclerosis	53 (57.6)	44 (55.0)	9 (75.0)	0.320
Small vessel occlusion	31 (33.7)	30 (37.5)	1 (8.3)	0.096
Cardioembolic	5 (5.4)	4 (5.0)	1 (8.3)	-
Other determined	-	-	-	-
Undetermined	3 (3.3)	2 (2.5)	1 (8.3)	0.850
**Posterior circulation stroke**	10 (10.9)	7 (8.8)	3 (25.0)	0.234
**Biological measures (, pg/ml)**				
CD40L	8.2 [5.3, 12.5]	8.2 [5.4, 12.5]	7.4 [5.3, 12.9]	0.953
HGF	90.1 [70.8, 126.9]	88.7 [69.4, 125.8]	106.1 [88.0, 154.3]	0.258
IL-1β	5.4 [3.6, 8.5]	5.6 [3.6, 9.3]	4.1 [3.5, 6.1]	0.268
IL-10	3.0 [1.9, 4.2]	3.1 [1.8, 4.2]	2.6 [2.2, 4.0]	0.902
IL-16	69.9 [47.0, 104.2]	60.6 [44.4, 96.8]	89.9 [74.8, 148.5]	0.010
IL-2	28.1 [18.4, 45.2]	28.7 [19.3, 46.2]	23.1 [18.3, 29.9]	0.266
IL-2Rα	953.6 [686.1, 1269.7]	947.6 [675.8, 1248.2]	1062.1 [911.4, 1498.2]	0.263
IL-5	66.5 [44.9, 108.1]	66.6 [42.4, 111.8]	57.8 [50.6, 75.4]	0.596
CCL20	15.8 [11.7, 20.5]	15.7 [11.5, 20.5]	16.2 [13.9, 19.9]	0.708
MMP1	5.6 [4.5, 7.2]	5.6 [4.7, 7.3]	4.7 [3.8, 5.8]	0.111

† $p < 0.05$.
[b] NIHSS score at 24h after rtPA treatment.

© 2022. Li L et al. Published online at http://www.aginganddisease.org/EN/10.14336/AD.2022.0608

[c] NIHSS score at 7 days after rtPA treatment, seven patients missing data.
Abbreviations: BMI = Body Mass Index; HbA1c = HemoglobinA1c; HDL = High density lipoprotein; HYC = Homocysteine; LDL = Low density lipoprotein; mRS = modified Rankin Scale; NIHSS = NIH Stroke Scale; NLR = Neutrophil-to-Lymphocyte Ratio; sICH = symptomatic intracerebral hemorrhage; TC = Total cholesterol; TG = Triglyceride; TOAST = Trial of ORG 10172 in Acute Stroke Treatment.
Data for continuous variables are described as mean (SD) (normally distributed variables) or as median [interquartile range] (nonnormally distributed variables), for categorical variables are described as n (%).

© 2022. Li L et al. Published online at http://www.aginganddisease.org/EN/10.14336/AD.2022.0608
